# Secular trends in height and weight among children and adolescents of the Seychelles, 1956–2006

**DOI:** 10.1186/1471-2458-8-166

**Published:** 2008-05-19

**Authors:** Pedro Marques-Vidal, George Madeleine, Sarah Romain, Anne Gabriel, Pascal Bovet

**Affiliations:** 1 Centre for Cardiovascular and Metabolic Research (Cardiomet), Lausanne, Switzerland; 2 Institute of Social and Preventive Medicine (IUMSP), University Hospital and University of Lausanne, Lausanne, Switzerland; 3 Unit for Prevention and Control of Cardiovascular Disease (UPCCD), Ministry of Health and Social Development, Victoria, Republic of Seychelles

## Abstract

**Background:**

Height of individuals has long been considered as a significant index of nutrition and health of a population; still, there is little information regarding the trends of height and weight among developing or transitional countries. We assessed the secular trends in height and weight in children of the Seychelles, a rapidly developing island state in the Indian Ocean (African region).

**Methods:**

Height and weight were measured in all students of all schools in four selected school grades (kindergarten, 4^th^, 7^th ^and 10^th ^grades) for the periods 1998–9 (6391 children) and 2005–6 (8582 children). Data for 1956–7 was extracted from a previously published report.

**Results:**

At age 15.5 years, boys/girls were on average 10/13 cm taller and 15/9 kg heavier in 2005–6 than in 1956–7. Height increased in boys/girls by 1.62/0.93 cm/decade between 1956–7 and 1998–9 and by 1.14/1.82 cm/decade between 1998–9 and 2005–6. For weight, the linear increase in boys/girls was 1.38/1.10 kg/decade between 1956–7 and 1998–9 and 2.21/2.50 kg/decade between 1998–9 and 2005–6. Overall, the relative increase in weight between 1956–7 and 2005–6 was 5-fold higher than the relative increase in height.

**Conclusion:**

Height and weight increased markedly over time in children aged <16 years in the Seychelles, consistent with large changes in socio-economic and nutritional indicators in the considered 50-year interval. The markedly steeper increase in weight than height over time is consistent with an epidemic of overweight and obesity.

## Background

Height of individuals has long been considered as a significant index of nutrition and health of a population [1,2]. In the last century, a consistent increase in mean height and weight has been found in children and adults, mirroring improvements in nutritional [3] and socio-economic status [4-9]. Although in Europe height has been increasing in most populations [9-12], some recent studies have reported that the increase in height has reached a plateau in Germany [13] or Poland [14]. This plateau was attributed to the fact that the corresponding populations had achieved their full genetic potential and/or that their socio-economic conditions had ceased to improve [13-15].

There is little information regarding the trends of height and weight among developing or transitional countries. Many African countries show no increase or even a decrease in height [1], albeit an increase was noted in some of them, e.g. Kenya and Senegal [1]. An increase in children height and weight was also reported for Brazil [16], Iran [17], Mexico [18], the Cook islands [7] and India [15]. Hence, studies of secular changes in height and weight in populations are useful for providing information on nutritional status in early life, updating growth reference standards, and providing insight with regards to epidemiological trends of cardiovascular disease [19,20]. Further, to our knowledge, little if no information is available regarding height and weight trends in Eastern Africa.

Thus, in this paper, we report the secular trends in height and weight among representative samples of children and adolescents of the Seychelles, a rapidly developing middle-income island state in the Indian Ocean (Eastern African region).

## Methods

### Study population

The Republic of the Seychelles is an archipelago located in the Indian Ocean, 1800 km east of the coast of Kenya and north of the island of Mauritius. Approximately 90% of the population lives on one island (Mahé) and most of the remaining population resides on two nearby islands. A large majority of the population is of African descent, with minorities of European, Indian, Chinese or mixed descent. The crude national gross domestic product per capita grew from US$600 in 1976 to US$8492 in 2004 [21], paralleling booming tourism and industrial fishing industries and a growing service-oriented economy. Cardiovascular disease accounts for 38% of all deaths [21] and high levels of cardiovascular risk factors have been documented in the adult population [22,23], as well as a marked increase in the prevalence of pediatric obesity [24].

### Sampling

For the surveys in 1998–9 and 2005–6, the sampling procedure and methods used data collection have been described previously [24]. Briefly, school nurses screen every year all children from four selected grades (kindergarten, 4^th^, 7^th ^and 10^th ^grade) in all public and private schools as part of an ongoing school-based health program. Nearly 100% of children attend school up to the 10^th ^grade with approximately 96% in public schools. A signed consent by the parents is sought for all children attending the screening program and students participate on a voluntary basis, with an overall participation rate of 77% [24].

For the study in 1956–7, the sampling procedures and methods used for data collection have been described previously [25]. Briefly, a representative sample of the entire population of Seychelles was obtained by systematic sampling every seventh house to gather a sample of around 5000–6000, which also corresponded to a seventh of the total population at that time. All inhabitants of all houses were eligible. In total, 984 houses were selected and 5587 persons of the 5766 inhabitants of the houses were examined, of which 982 boys and 1043 girls aged between 5 and 20 years. In this study, data corresponding to the following ages was used: 5 to 9, 11 to 13 and 15 years. As the data collected in 1956–7 used only completed years and not decimal years, we decided to use the same methodology as for the other surveys and the mid-point was used to define age (i.e. 6.5 years for children aged between 6.0 and 6.9 years).

### Height and weight data

For the studies in 1998–9 and 2005–6, height and weight were measured with fixed stadiometers (Seca 208) and precision electronic scales (Seca 870, Seca, Hamburg, Germany), respectively. Children were measured without shoes and in light clothing. School nurses were regularly trained on the measurement techniques to ensure consistency over time and between schools. Weighing scales and stadiometers were checked for accuracy at regular intervals. Body mass index (BMI) was calculated as weight divided by height squared (kg/m^2^). In this study, we pooled data for surveys available between 1998 to 2006 within periods of 2 consecutive years to increase the stability of the estimates (students examined over two subsequent years are different). We used the first and latest available periods, i.e. 1998–9 and 2005–6. Obesity levels were assessed using the International Obesity Task Force recommendations [26].

For the period 1956–7, all the measurements were made at the houses of the participants [25]. Height was measured, without shoes, to the nearest eighth of an inch for children up to the age of 16 years. A special height recorder with a wooden platform was used. Weight was measured to the nearest half pound for children with only minimum clothing. A bathroom scale was used, which was daily checked for accuracy over its whole range.

Growth data was also assessed for 2003. This year was chosen since the school survey was extended to a random sample of classes of fifth grade of secondary schools (not compulsory, but attended by >90% of all children) and of first year post secondary schools (both academic and vocational). In 2003, there were thus 5,793 students in kindergarten, 4^th^, 7^th ^and 10^th ^grade (i.e. within the usual school surveillance system) and 1,236 additional students in the 11^th ^and 12^th ^grades aged 17,9 ± 1.0 (mean ± SD).

### Statistical analyses

Statistical analyses were conducted using Stata v9.2 for Window (Stata Corp, Texas, USA) and SAS (SAS, Cary, USA). Secular trends were assessed by weighted linear regression on mean values of height and weight computed for each survey, age and gender. Typically, in this method, the observations represent averages and the weights are the number of elements that gave rise to the average. The relationships between age and height or weight were assessed separately for each gender using a general linear model adjusting for study period. Between-period comparisons of height and weight were performed separately for each gender/age pair using Student's t-test. Results were expressed as 10-year increase in height or weight and [95% confidence interval], or as relative increase using the first period as reference. Two intervals were considered: 1956–7 to 1998–9 (average: 42 years) and 1998–9 to 2005–6 (average: 7 years). Norms of height, weight and body mass index in 2003 are based on trends over age calculated separately for boys and girls using polynomial equations of third of fourth degrees. In a few instances, the value of height, weight or body mass index for the lowest age category (e.g. age 5) has been adjusted to not be larger than the value of the next older age category and the value of the highest age category (e.g. age 19) adjusted to not be smaller than the value of the previous (younger) age category: in all instances changes are less than 1% in absolute value. Due to the number of statistical tests performed, statistical significance was considered for two-tailed p < 0.01.

## Results

### Secular trends

**Figure 1:**
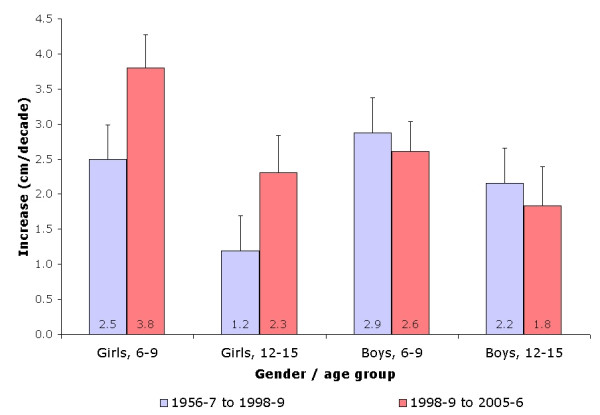
Increase in body height (in cm per decade) according to gender, age group and study period.

**Figure 2:**
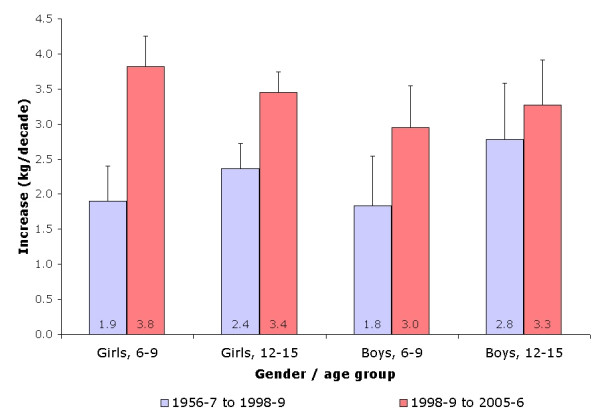
Increase in body weight (in kg per decade) according to gender, age group and study period.

The secular trends in height and weight according to gender are summarized in figures 1 and 2. In boys, the mean increase in height was 1.62 cm per decade (range: 1.18 to 2.93 cm/decade depending on age group) for the period 1956–7 to 1998–9, and 1.14 cm per decade (range: 0.06 to 4.86 cm/decade) for the subsequent period (1998–8 to 2005–6). The increase in height tended to be higher among boys aged 9.5 to 12.5 years for the first period, and among boys aged 12.5 to 13.5 years for the second period (figure 1). In girls, the mean increase in height was only 0.93 cm per decade (range: 0.44 to 3.16 cm/decade depending on age group) for the period between 1956–7 and 1998–9, and almost the double afterwards: 1.82 cm per decade (range: 0.12 to 2.73 cm/decade).

The increase in height tended to be higher among girls aged 12.5 to 13.5 years in both periods (figure 1). On a relative basis, children in 1998–9 were 7% (boys) to 5% (girls) taller than those in 1956–7, and a further 1% increase was found among children surveyed in 2005–6 relative to those surveyed in 1998–9.

Regarding weight, the mean increase in boys was 1.38 kg per decade (range: 0.7 to 3.49 kg/decade depending on age group) for the period 1956–7 to 1998–9, and 2.21 kg per decade (range: 0.10 to 5.51 kg/decade) for the subsequent period, while the corresponding figures in girls were 1.1 kg (range: 0.63 to 3.13 kg/decade) and 2.5 kg per decade (range: 0.44 to 4.43 kg/decade). In boys, the strongest increases were noted for the children aged over 11, for the first period (>2.0 kg/decade), and for the children aged between 11 and 13 years for the second period (>4.0 kg/decade), while for girls the strongest increases were found in the participants aged between 11 and 13 years (figure 2). On a relative basis, the children surveyed in 1998–9 were 28% heavier than those surveyed in 1956–7, and a further 5% increase was found among the children surveyed in 2005–6 relative to those surveyed in 1998–9.

Overall, the relative increase in weight over calendar years was 5-fold higher than the relative increase in height, and was paralleled by an increase in obesity levels from 2.7% in 1998–9 to 5.2% in 2005–6 in boys (p < 0.001), the corresponding values in girls being 4.2% and 7.3% (p < 0.001).

### Increase in height and weight with age

The relationships between anthropometric markers and children's age according to gender and study period are summarized in table 1. Overall, in both genders, no significant differences were found between study periods regarding the increase in height and weight. It is worth noting that, although the overall comparison between the three study periods for weight gain with age was not significant, the weight gain with age observed for 2005–6 in boys was significantly (p < 0.05) higher than for 1956–7 (table 1).

**Table 1:** Relationships between anthropometric markers and children's age according to gender and study period

	**1956–7**	**1998–9**	**2005–6**	**ANCOVA**	**P value**
*Boys*					
Height (cm/year of age)	5.17 [4.86–5.48]	5.51 [5.15–5.87]	5.54 [5.30–5.79]	0.67	0.526
Weight (kg/year of age)	2.44 [2.02–2.86]	3.61 [2.97–4.25]	3.80 [3.38–4.22]	2.89	0.081
*Girls*					
Height (cm/year of age)	5.24 [4.96–5.53]	5.05 [4.11–5.99]	5.06 [4.01 – 6.12]	0.02	0.983
Weight (kg/year of age)	2.86 [2.20–3.53]	3.63 [3.22 – 4.04]	3.75 [3.27–4.24]	1.66	0.219

Results are expressed as increase (in height, respectively weight) per year of children's age (i.e. slope) and [95% confidence interval] calculated from the pooled data of the two years of each period. ANCOVA: test for equality of the slopes between periods.

**Figure 3:**
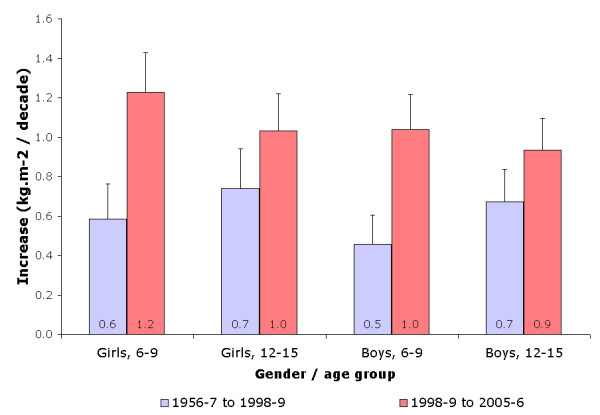
Increase in body mass index (in kg/m^2 ^per decade) according to gender, age group and study period.

Finally, there was an acceleration in the decennial increase of weight and BMI in both genders and age groups between study periods (figures 2 and 3). Conversely, for height, an acceleration was found in girls for both age groups, whereas no differences were found for boys (figure 1).

Table 2 provides sex and age specific percentiles of height, weight and body mass index in boys and girls aged 5 to 19 years in 2003. While the distribution of height is fairly normally distributed in all age categories, the distributions of weight and body mass index are highly skewed to high values and increasingly so as age increases. Height plateaus at the age of 14–15 years in girls and the age of 18–19 years in boys.

**Table 2:** Age and sex-specific percentiles of height, weight and body mass index in children and adolescents aged 5–19 years in 2003

	**Boys**							**Girls**						
	**P5**	**P10**	**P25**	**P50**	**P75**	**P90**	**P95**	**P5**	**P10**	**P25**	**P50**	**P75**	**P90**	**P95**
**Height (cm)**														
5	104	105	108	111	114	117	118	102	104	107	110	114	117	119
6	108	109	112	116	119	123	124	106	107	111	114	118	122	124
7	112	114	117	121	125	129	131	111	113	116	120	124	128	131
8	117	119	123	127	131	135	137	117	119	123	126	131	135	139
9	123	125	129	133	137	142	144	124	126	130	134	138	143	146
10	128	131	135	139	144	148	151	130	133	137	141	145	150	153
11	134	136	140	145	150	155	157	136	139	143	147	152	156	159
12	139	142	146	151	156	161	163	141	144	148	152	157	161	164
13	145	147	152	157	162	166	169	145	148	152	156	161	165	168
14	150	152	157	162	167	171	174	148	150	154	159	164	167	170
15	154	157	161	166	172	176	179	150	152	156	160	165	168	171
16	158	160	165	170	175	179	182	151	152	156	161	165	169	172
17	161	164	168	173	178	182	185	151	152	157	160	165	169	171
18	163	166	170	175	179	183	186	151	152	157	160	165	169	171
19	164	167	171	175	180	183	186	152	153	159	161	166	169	172
**Weight (kg)**														
5	16	16	18	19	20	21	23	15	16	17	19	20	22	23
6	16	17	18	19	22	26	29	15	16	17	19	21	24	29
7	17	18	19	21	25	31	34	16	17	18	21	24	28	37
8	19	19	21	24	28	36	40	19	20	21	24	29	34	44
9	21	22	24	27	32	41	46	21	23	25	29	35	42	50
10	23	24	27	31	37	46	52	25	26	29	34	41	50	56
11	26	28	31	35	41	51	58	28	30	34	39	46	56	61
12	29	31	35	39	46	56	63	31	33	38	43	51	62	66
13	33	35	39	44	50	60	69	34	36	41	47	55	66	71
14	36	38	43	48	55	65	74	36	39	44	50	58	69	74
15	40	42	47	52	59	69	79	38	41	46	52	60	70	77
16	43	46	51	56	63	73	83	40	43	48	54	61	70	79
17	47	49	54	60	67	77	86	41	44	49	54	60	69	80
18	50	52	57	63	70	81	89	42	45	49	54	60	69	81
19	53	55	59	65	73	84	90	43	46	49	54	60	70	81
**Body mass index (kg/m^**2**^)**														
5	12.5	13.1	14.0	14.7	15.5	17.4	18.2	12.5	12.9	13.6	14.6	15.7	16.9	18.0
6	12.5	13.1	14.0	14.8	16.0	18.4	19.5	12.5	13.0	13.7	14.7	16.3	18.4	19.8
7	12.5	13.1	14.0	14.9	16.5	19.3	20.7	12.5	13.1	13.8	15.0	17.1	19.8	21.5
8	12.6	13.2	14.1	15.2	17.0	20.1	21.9	12.7	13.2	13.9	15.4	17.9	21.1	23.0
9	12.8	13.1	14.4	15.5	17.5	20.9	22.9	12.9	13.6	14.4	16.0	18.7	22.3	24.4
10	13.2	13.7	14.7	16.0	18.0	21.7	23.9	13.3	14.0	15.0	16.7	19.5	23.3	25.7
11	13.6	14.1	15.1	16.5	18.5	22.4	24.8	13.7	14.5	15.6	17.5	20.4	24.2	26.7
12	14.1	14.5	15.6	17.1	19.1	23.0	25.6	14.2	15.0	16.3	18.3	21.2	25.0	27.7
13	14.6	15.0	16.1	17.7	19.7	23.6	26.3	14.7	15.6	17.0	19.1	21.9	25.6	28.5
14	15.1	15.6	16.7	18.3	20.2	24.2	27.0	15.3	16.1	17.5	19.8	22.5	26.2	29.2
15	15.7	16.1	17.3	18.9	20.8	24.7	27.5	15.8	16.7	18.0	20.3	23.0	26.6	29.7
16	16.2	16.7	17.8	19.4	21.4	25.1	28.0	16.3	17.2	18.4	20.7	23.3	26.9	30.0
17	16.7	17.3	18.4	19.9	22.0	25.5	28.4	16.8	17.6	18.6	20.8	23.3	27.0	30.3
18	17.1	17.8	19.0	20.3	22.3	25.8	28.8	17.1	17.9	18.7	20.8	23.4	27.0	30.4
19	17.3	18.3	19.5	20.6	23.3	26.1	29.0	17.4	18.0	18.7	20.9	23.4	27.1	30.4

Values correspond to the exact age mentioned (e.g. age 5 stands for age 5.0)

## Discussion

To our knowledge, this is the first report on secular trends among children and adolescents of the African region. In this study, a secular upward trend for height and weight among children and adolescents of the Seychelles could be demonstrated. For instance, at age 15.5 years, boys/girls were on average 10/13 cm taller and 15/9 kg heavier in 2005–6 than in 1956–7.

In most countries for which trends in children and adolescents' height and weight have been published, a secular increase has been noted [16,27-33], although some studies have reported a stabilization [13,14] or even a downward trend [34], likely due to unfavorable conditions such as war [17] or economical crisis [34]. In the Seychelles, the upward trends in height and weight were comparable in magnitude to those found in other countries, i.e. the USA [35], Australia [30], Brazil [7] and Turkey [33] and paralleled the increase in the *per capita *national gross domestic product, which rose in real terms from US$ 2927 in 1980 to US$5239 in 2004 [21].

The secular trends in height and weight can be affected by nutritional [3] and socio-economic factors [5,7,8], although this last statement has been challenged [36]. Other factors such as earlier maturation have also been suggested [27,30,37,38]. Indeed, data from food balance sheets indicate that the per capita calorie availability has increased substantially in Seychelles, from 1800 kcal in 1965 to 2300 kcal in the late 1980s, and above 2400 kcal in the early 2000s [39]. The proportion of carbohydrates has decreased over time (74% of total calories in 1965 and 55% in 2000) while the proportion of fats has increased (16% in 1965 and 32% in 2000) [39]. Also, according to households expenditure surveys, between 1983 and 1993 the consumption of meat products increased by 238% in the Seychelles, while the consumption of fish, fruits and vegetables decreased by 33% [40]. Thus, it is likely that the improvements in socio-economic factors and the changes in food intake and in physical activity levels which occurred in the last decades in the Seychelles might partly explain the increases in height and weight observed in this study, although other factors cannot be ruled out. For instance, in the study conducted in 1956–7, a significant percentage of children presented with stunting [25], a very rare condition in the Seychelles nowadays. As the height of children is influenced by their parents' height, the fact that in the 1950s a substantial proportion of now-become parents were stunted might have reduced the increase in their children's height and weight, irrespective of a current good nutrition environment. Finally, some authors have suggested that secular trends in growth are more responsive (or plastic) to changes in the environment in boys than in girls [4,41]. This might partly explain the higher increase in height in boys in the first period (1956–7 to 1998–9) of this study.

It has also been described that secular trends in height observed in young children might be carried over into adulthood [42]. Although data on adult height in the Seychelles is not available for the 50-year period under study, two types of information can help address indirectly this issue. First, in girls, the secular increase in height was much smaller at the age of 16 years than for the younger age groups (figure 1); for boys (figure 2) the results are inconclusive as the rapid growth period during puberty typically ends after the age of 16, for which no data is available for comparison over time in this study. Second, based on two population surveys in adults [22,23], height at age 25–34 years was virtually identical in 1989 and 2004. Overall, these findings support the hypothesis that at least part of the increase in height in children during the past decades reflects accelerated growth during childhood over successive cohorts *without *a commensurate increase in height in adulthood, i.e. children grew faster during childhood but reached similar height after completion of adolescence, a trend also reported for Greenlandic children [27] and for India, where after a significant positive secular trend in height attained over the first 20 years, the adult height has now plateaued [15]. Finally, it is of interest to note that, in girls, the increase in height per decade was higher for period 1998–9 to 2005–6 than for period 1956–7 to 1998–9, suggesting an acceleration, a feature also reported in Mexico [18], where no secular change in height was noted among primary school children between 1968 and 1978, with a significant increase for period 1978–2000.

Weight increased over time at a much larger pace than height. These findings are in agreement with a marked secular increase in BMI reported in the Seychelles [24] and in other countries [43-46], with some exceptions [47]. This larger increase over time in weight than height might reflect a larger increase over time in adiposity relative to muscle mass [29,48]. Indeed, the stronger increase in weight relative to the increase in height led to an increase in BMI and the prevalence of obesity among Seychellois children and adolescents. The upward trends in weight and BMI are partly associated with a concomitant decrease in levels of physical activity over time, as shown in Seychelles [24] although data on the relationship between lack of physical activity and overweight are still inconclusive [49]. Still, as no data for physical activity is available for the 1956–7 period, it was not possible to assess the possible impact of changes in physical activity levels on the increase in weight. Also, the effects on weight of an increased energy intake [39,40] or of improvements in health services such as a decrease in intestinal parasites among children from 95% in 1956–7 [25] to less than 15% in 1997 [50] cannot be ruled out. Overall, these findings are consistent with a shift from substantial under nutrition in children in 1956–7 to an ongoing epidemic of overweight/obesity in the youth population of the Seychelles.

The observed secular increases in height and weight stress the need to adequately update reference growth curves. Indeed, many reference growth curves in children have been developed several decades ago and might no longer reflect the current distribution of body height and weight [29,32,33,51]. However, if updates in height norms can be validly and simply carried out based on current data, the update in weight norms, using current data, is more problematic as many countries currently face an increase in pediatric obesity [52], inclusive Seychelles [24]. Deriving weight standards from the current anthropometric data would artificially decrease the prevalence of obese children and adolescents. Thus, the sex-and age- specific cut off values for overweight and obesity should be established carefully for the pediatric population and it has been advised that universal internationally agreed-upon thresholds be used [53]. Further, increased height in current vs. previous generations of children may have important implications with regards to the measurement and interpretation of indicators that are related to height. For example, cut off values for high blood pressure, which relate strongly with a child's age and height [54], might need to be adjusted if children of same age are taller nowadays than before.

This study has some limitations. First, it was not possible to assess individual data for the period 1956–7, and only average age-specific values were available for comparison. Still, this method has been used by others in order to assess trends for long time periods [14,32]. Second, no data on pubertal status was collected, preventing the possibility of assessing whether pubertal age has decreased over time. Third, data available for comparison between time periods were limited to children aged <16 years, which prevented to directly examine if the observed height gain in children over successive cohorts tracked into adulthood. Fourth, estimations are only based on 3 cross-sectional surveys over the whole study period, and the period 1998–2006 might be too short to properly trends; further, the increase in anthropometric measurements might be non-linear, but as only three time points were available, a non-linear model could not be applied. Still, as the yearly assessment of height and weight is currently under way, a better trend assessment will be achievable within some years. Also, it will be possible to follow the cohorts of children, thus leading better estimates of growth curves. Finally, the main strengths of this study are the long time interval examined (50 years), the large size of the samples (particularly for 1998–2006), the population-based nature of the data in all periods, and the use of standardized measurements throughout the study period (particularly for 1998–2006).

## Conclusion

In summary, a secular upward trend in body height was documented in children and adolescents of both genders in the Seychelles and the rate of increase was not different during the last decade vs. the previous 4 decades. Indirect evidence suggests that this upward height gain in youth over time may not have translated in a commensurate height gain in adults and further studies should be conducted to fully assess the significance of such upward height trends among youth. Conversely, the markedly steeper upward secular trend in weight than height suggests that the young population of the Seychelles is experiencing an epidemic of overweight and obesity.

## Competing interests

The authors declare that they have no competing interests.

## Authors' contributions

PM–V lead the data analysis and the write up of the manuscript. GM managed the database of children in 1998–2006 and reviewed the paper. SR and AG assisted in the interpretation of data and reviewed the manuscript. PB assisted with data analysis and the write up of the manuscript. All authors read and approved the final manuscript.

## Pre-publication history

The pre-publication history for this paper can be accessed here:


